# Conflict before the courtroom: challenging cognitive biases in critical decision-making

**DOI:** 10.1136/medethics-2020-106177

**Published:** 2020-07-06

**Authors:** Harleen Kaur Johal, Christopher Danbury

**Affiliations:** 1 Centre for Ethics in Medicine, University of Bristol, Bristol, UK; 2 Adult Intensive Care Unit, Royal Berkshire NHS Foundation Trust, Reading, UK; 3 School of Law, University of Reading, Reading, UK

**Keywords:** ethics, end of life care, capacity, decision-making, psychology

## Abstract

Conflict is an important consideration in the intensive care unit (ICU). In this setting, conflict most commonly occurs over the ‘best interests’ of the incapacitated adult patient; for instance, when families seek aggressive life-sustaining treatments, which are thought by the medical team to be potentially inappropriate. Indeed, indecision on futility of treatment and the initiation of end-of-life discussions are recognised to be among the greatest challenges of working in the ICU, leading to emotional and psychological ‘burnout*’* in ICU teams. When these disagreements occur, they may be within the clinical team or among those close to the patient, or between the clinical team and those close to the patient. It is, therefore, crucial to have a theoretical understanding of decision-making itself, as unpicking misalignments in the family’s and clinical team’s decision-making processes may offer strategies to resolve conflict. Here, we relate Kahneman and Tversky’s work on cognitive biases and behavioural economics to the ICU environment, arguing that these biases could partly explain disparities in the decision-making processes for the two conflicting parties. We suggest that through the establishment of common ground, challenging of cognitive biases and formulation of mutually agreeable solutions, mediation may offer a pragmatic and cost-effective solution to conflict resolution. The litigation process is intrinsically adversarial and strains the doctor–patient–relative relationship. Thus an alternative external party should be considered, however mediation is not frequently used and more research is needed into its effectiveness in resolving conflicts in the ICU.

## Introduction

Conflict is an important consideration in the intensive care init (ICU).^1^ With lengthening average life expectancies and widening availability of life-sustaining interventions; the question of when treatment becomes medically ‘futile’ (i.e. incapable of generating the desired physiological result) or inappropriate, is becoming increasingly difficult to answer.^2^ Advanced care planning in healthy individuals is infrequent,^3^ thus little consideration is given to whether a person would deem different treatment options to have a meaningful impact on their quality or length of life, while the person is able to communicate these views.^4^ These conversations consequently arise when the person is no longer able to participate in the decision-making process, due to the severity of their illness. In England and Wales, doctors frequently determine the course of medical treatment, based on what they reasonably believe to be best for the incapacitated patient. Families, who are consulted in this process, may feel accountable for making difficult choices from the imagined perspective of a patient; to either seek aggressive, life-sustaining treatments or focus on symptom management at the end-of-life.^2^ Interestingly, empirical evidence suggests that independent elderly individuals are reluctant to accept life-sustaining treatments such as invasive mechanical ventilation and renal replacement therapy, as they can negatively affect quality of life.^4 5^ These findings have recently been validated across Europe.^6^ However, conflict most commonly occurs when families seek aggressive life-sustaining treatments that are thought, by the medical team, to be potentially inappropriate.^4^ The international ‘Conflicus’ study surveyed the experiences of 7498 ICU staff members in 323 ICUs. Nurse–physician conflicts were most common (32.6%) and staff–relative conflicts accounted for 26.2% of perceived conflicts.^1^ Indecision on futility of treatment and the initiation of end-of-life discussions are recognised to be among the greatest challenges of working in the ICU, leading to emotional and psychological ‘burnout’ in ICU teams.^7^ Given its harmful effects, a deeper understanding of why conflict occurs is necessary, alongside potential strategies to resolve it.

## When does conflict occur?

Conflict is taken to mean a ‘a serious disagreement or argument, typically a protracted one*’*. The term conflict is often used interchangeably with ‘dispute’, however, Burton and Dukes offer a distinction between the two, based on timescale and the negotiability of the issues at hand. Conflicts may involve long-term, non-negotiable issues whereas disputes are short-term and usually quickly resolved.^8^ In the ICU, conflicts frequently relate to entrenched disagreements over decisions being made in a patient’s ‘best interests*’*.

As per the Mental Capacity (MCA) 2005 in England and Wales, most adult patients in the ICU are incapacitated due to either the effects of sedation, which is necessary for organ support, or the encephalopathy associated with critical illness. This results in ‘an impairment of or disturbance in the functioning of [their] mind or brain*’*,^9^ which prevents a person from understanding, retaining, deliberating or communicating healthcare decisions; therefore inferring they lack capacity to make these decisions. Principle 4 of the MCA states that where a person has been assessed as lacking capacity, any decision made for or on behalf of that person should be made in their best interests. Indeed, the best interests standard applies to incompetent or incapacitated persons of all ages in many international jurisdictions.^10^ There is no definition in the MCA, or Code of Practice, about who this ‘decision-maker*’* is.^9 11^ In the context of ICU, when the patient does not have an advance decision to refuse treatment (ADRT) or a lasting power of attorney (LPA) for Health and Welfare decisions, the decision-maker, in practice, is the consultant intensivist with clinical responsibility for the incapacitated patient.^12^ (Similar instruments and roles exist internationally, such as Advance Directives and Durable Powers of Attorney in the USA).^13^ Any decisions made in the patient’s best interests, should achieve the purpose that is being sought, in the way that is least restrictive of their rights and freedoms.^9^ A person can write an ADRT, which — assuming it is valid and applicable — is determinative with regard to refusal of the specified future treatments.^9^ If this document exists and there is no doubt of its legality or intention, it must be respected. If an ADRT is not available or applicable, the decision-maker must take into account anyone previously named by the patient as someone to be consulted, and anyone engaged in caring for the person or interested in their welfare.

Conflicts may occur when there is a prolonged disagreement about where the best interests of the patient lie. For instance, when families favour preservation of life and physicians are reluctant to provide aggressive treatment, which they deem to be inappropriate. Nevertheless, it is worth acknowledging that a treatment may still further the patient’s reasonable goals of care, even if it is not curative or palliative, and it is not merely inappropriate because it has a low probability of success.^14^ Ultimately, conflict could arise in any healthcare setting, in any jurisdiction. Thus, the discussion around best interests conflicts in ICUs could be extrapolated to other conflicts between healthcare teams and representatives of the incapacitated (or incompetent) patient.

## Why does conflict occur?

When there is contention about the patient’s best interests, conflict can develop. The disagreement may be within the clinical team or among those close to the patient, or between the clinical team and those close to the patient. It is therefore crucial to have a theoretical understanding of decision-making itself. Unpicking misalignments in the family’s and physician’s decision-making processes may offer strategies to resolve conflict. Although the adult ICU is given as the primary example, these ideas are broadly applicable to other specialties, such as paediatric intensive care, palliative care, and care of the elderly.

Acclaimed theories in cognitive psychology and behavioural economics suggest how people make decisions. Kahneman and Tversky’s ‘prospect theory’ states that individuals make decisions based on the potential value of losses and gains, rather than the final outcome. A decision-maker will determine what outcomes are equivalent and set these as a ‘reference point’. The economic interpretation of this theory sets current assets or the status quo as a reference point, however, this can be readily applied to choices involving other attributes. In healthcare one can set their current quality of life as a reference point. Lesser outcomes in relation to this reference point are classified as ‘losses’, whereas greater outcomes are classified as ‘gains’. Prospect theory also offers the concept of ‘loss aversion’. Simply phrased, this is the idea that losses hurt more than gains feel good. Loss aversion is relative to expectations however, as an expectation of a great loss may lead to the interpretation of only a small loss as a gain.^15^ Implementing this in the healthcare setting, Kahneman and Tversky suggest that a person will make a decision to avoid losses, thus the decision-making process of a patient’s family members will be influenced by their desire to avoid the loss of their relative. Furthermore, if they are expecting their relative to die, they may interpret loss of quality of life as a comparative gain. Loss aversion, as an observed phenomenon, favours stability over changes in the status quo.^16^ This may explain why relatives continue seeking life-prolonging treatments for their relative, at the expense of quality of life. This could also be explained by another observation noted by Kahneman and Tversky. They suggested that a person who had not made peace with their financial losses, would be more likely to accept gambles that would otherwise be unacceptable to him or her. Similarly, relatives who have not yet accepted the patient is dying, may be keen to attempt treatment options with small chances of success, including those considered by the medical team to be potentially inappropriate. Another well-established theory states that an individual’s tendency to bet on ‘long shots’ increases throughout the betting day. If the betting day is taken to be the length of an ICU admission, families may wish to seek treatments with considerable risk or minimal chances of success, longer into the admission.^15^ Certainly, the idea of risk-seeking in losses was observed to be a robust effect in several psychological experiments. A large majority of people expressed a preference for the gamble over the secure loss. Hence, family decision-makers may insist on improbable treatment options, where the alternative would be guaranteed loss of their relative.^16^

Kahneman and Tversky also propose how a reference point is set, suggesting that the past and present context of experience defines an individual’s adaptation level. Thus, external events are perceived in relation to this reference point. For instance, a certain level of health may imply disability for one person and great health for another, depending on their previous experiences of illness. As critics of normative models of decision-making, particularly Bernoulli’s ‘expected utility theory’,^17^ Kahneman and Tversky offered a descriptive model whereby focus lay on people’s beliefs and preferences. They argue that humans do not naturally make logical or rational choices but instead, the decision-making process is framed by ‘cognitive biases’. This is secondary to a tendency to oversimplify decisions by basing their understanding of the decision on their previous experiences, which are probably unrepresentative sets of observations. Hence, Kahneman suggests that the mind primarily deals with ‘known knowns’, which are its observations. It rarely considers ‘known unknowns’, which are recognised as relevant but do not factor significantly into the decision-making process, due to a lack of experiential information. The human mind is therefore oblivious to ‘unknown unknowns’, i.e. occurrences, of which the mind has no prior knowledge or experience.^18^

Anecdotally, families insist that their relative ‘is a fighter’ or ‘has been here before’, when they have previously survived other bouts of illness or psychosocial ordeals.^19^ This can fuel a desire to persist with aggressive therapy, as family members hold the distorted assumption that future outcomes will mirror the past. Likewise, this concept can be applied to physicians. With 10%–29% of all ICU admissions ending in mortality,^20^ the decisions of ICU clinicians are more likely to be influenced by the relative normalisation of death as an outcome for critically unwell patients. Their reference point with regard to the patient is also likely to be lesser than a relative’s, as their only encounter with the patient is during a period of severe illness. This highlights the importance of determining a person’s functional baseline, when determining treatment options. Death is at risk of being perceived as less of a loss by physicians than it would be for the relative, whose reference point is of a well person.^21^ Thus, the variance in decision-making could be explained by disparities in the prior experiences and knowledge of the two conflicting parties. A difference in emotional ties to the patient is also of obvious importance, however the decision-maker’s psychological frame of mind is not accounted for in prospect theory. This shortcoming has been recognised, as critics of Kahneman and Tversky’s work cite their lack of recognition of emotion as one of the failings of prospect theory as a descriptive model of decision theory.^22^

Several psychological experiments have defined other cognitive biases, or pitfalls in the rationality of decision-making, which do account for emotions. An example is ‘confirmation bias’: the tendency to search for information in such a way that confirms pre-existing beliefs.^23^ This bias is noted to have a stronger effect in the context of emotionally charged issues. It becomes apparent in ICU, when families display a tendency to seek information that validates their belief that their relative has a good chance of survival. This can be compounded with another observed psychological phenomenon known as ‘the boomerang effect’, where attempts to persuade an individual of a certain view leads to the unintended adoption of the opposing position instead. Hovland *et al* noted this was more likely to occur in situations where the attempts at persuasion trigger unremitting emotional distress (as in the case of withdrawing life-sustaining treatment) or when the communicator’s position is far removed from the recipient’s (as in the case of a doctor–relative relationship).^24^ This may explain why attempts by ICU clinicians to convince relatives to withdraw life-sustaining treatment can paradoxically cement their view to continue with such treatment, leading to intractable disputes. Equally, rather than negatively responding to the physicians’ response, one may hold a (falsely) optimistic view of the potential outcome. ‘Optimism overconfidence’ here describes the phenomenon where an individual believes that he or she is less likely to experience a negative event. Hence, his or her position will be based on an unrealistic analysis of the potential outcomes.^25^ A relative may assume that the patient is less likely to die, due to their perception of the situation. However, optimism bias occurs more often when people perceive themselves to have greater control over events than other people (e.g. ‘I am less likely to crash a car if I am the driver*’*), which is not the case in an ICU setting. Indeed, the opposite can hold true and one may be less optimistic if they have prior experience of similar events.^26^ Thus, ICU clinicians, who encounter withdrawal of treatment decisions regularly, may possess less optimistic views of the potential outcomes, guided by evidence-based prognostication.^27^ This leads to further divergence between the views of families and ICU clinicians.

## What should we do when conflict arises?

When a disagreement occurs over the treatment of an incapacitated patient, hereafter referred to as P, a decision must somehow be reached. When P’s wishes are clearly known, through an ADRT or LPA (or their international equivalents), one must defer to those wishes. If a best interests decision must be made, one cannot routinely accept the clinical team’s decision in isolation, as the best interests principle within the MCA 2005 clearly states that the views of those close to P must be considered. Conversely, if one adopts the family’s point of view as default, this negates the clinical team’s expertise and previous experience. While the right to self-determination provides a negative freedom to refuse treatment, as entitled by the principle of autonomy, this cannot be adapted into a positive freedom to demand life-sustaining interventions (despite the wishes of the patient or family) as was demonstrated in *R (Burke)*.^28^ This leaves a final option: to involve a third-party.

In England and Wales, recent case law has encouraged parties to seek alternative dispute resolution methods, the learnings from which can be applied to various healthcare settings. In *Re: Y*, Lady Black reinforced a reasonable course of action for determining the best interests of a patient with a prolonged disorder of consciousness. In her judgement, she emphasised the involvement of a senior independent clinician in the decision-making process, to ensure that ‘the interests of patients and their families are safeguarded, as far as possible, against errors in diagnosis and evaluation, premature decisions, and local variations in practice*’*.^29^ For P’s relatives, however, another doctor might not be seen as a neutral or independent party. If the second medical opinion aligns with the first physician’s, it may even reinforce the conflicting views of the involved parties, as per the previously described boomerang effect. Thus, it may help to safeguard the interests of the patient but it will not necessarily benefit the doctor-patient-relative relationship. Lady Black’s judgement confirmed that ‘if the provisions of the MCA 2005 are followed and the relevant guidance observed, and if there is agreement on what is in the best interests of the patient, the patient may be treated in accordance with that agreement without application to the court*’*.^29^ If a consensus cannot be reached on the best interests of P, an application to the court can and should be made. Given that litigation is universally a costly process, both emotionally and financially, it can reasonably be described as a last resort.

Increasing attention has since been given to other ways of resolving conflict, before it reaches the courtroom. In the recent *Gard* case, where a conflict occurred between the physicians and family caring for a young child, Justice Francis suggested that ‘mediation should be attempted in all cases such as this one even if all that it does is achieve a greater understanding by the parties of each other’s positions’.^30^ Mediation is a non-adjudicative process that has long been used as a form of alternative dispute resolution throughout the world,^31 32^ notably where legal proceedings relate to disputes of money or the welfare of children. As children have always been recognised as the ‘incapacitated’ fraction of society, having (below the age of sixteen) to prove their competence (unlike adults, where capacity is assumed), they too have always had their best interests determined by proxy decision-makers. However, with an increasing prevalence of age-related incapacitating disease, it is important to extend these principles to the elderly population. A comprehensive Canadian report on ‘elder law’ aimed to appraise mediation as a method for resolving disputes in the care of the elderly population, where the elderly person is still conscious and presumably able to participate in the decision-making process (although with questionable capacity). It emphasised the vital role of mediation in dispute resolution between families and healthcare providers, around the best interests of P.^33^

Mediation itself is difficult to define, as it has several approaches. However, it is described by Mary Radford as:

A process in which an impartial third party — a mediator — facilitates the resolution of a dispute by promoting voluntary agreement (or ‘self-determination’) by the parties to the dispute. A mediator facilitates communications, promotes understanding, focuses the parties on their interests, and seeks creative problem-solving to enable the parties to reach their own agreement.^31^

No formal model of mediation exists, as mediators come from a variety of professional backgrounds with varying experience. However, the Canadian report concluded that a successful mediator in these cases requires a sound understanding of both medicine and the law.^33^ The overarching premise of mediation is to reunite the two conflicting parties, with each party advocating their own interests, before reaching a mutually agreeable decision about P. This contrasts to litigation, where one of the two conflicting parties is ultimately set to ‘lose’, thus securing the wedge between them (see table 1).^34^ A key approach in mediation is to challenge the cognitive biases, or ‘de-bias’, the two conflicting parties.^35^ By dispelling confounders in the decision-making process for both parties, it is possible to establish common ground and resurface the central issue, which is the determination of P’s the best interests.

**Table 1:** A comparison of mediation and litigation methods^42^

Mediation	Litigation
Consensual	Adversarial
Inexpensive	Costly
Confidential	Part of public record
Formulation of mutually agreed solution between conflicting parties	Decisions made by external party (judge)
Time-scale determined by conflicting parties	Time-consuming
Less regulated	Subject to formal review
A mutually agreed solution may not be made	Necessarily leads to resolution

Critics of mediation raise concerns around its regulation. While litigation is well established and subject to formal review, mediation relies on the fairness and ability of the trained mediator, often in a private setting. This objectivity is a fundamental ethical consideration in mediation and relies on the mediator’s own ethical code of practice.^31^ Maintaining a neutral position also allows the mediator’s primary aim to be achieved: to facilitate, not impose, a decision that is acceptable to both parties. A partial answer to this concern is that in cases issued in the Court of Protection, any agreement reached through mediation about P’s best interests would need to be reviewed by the Court. This clearly would not apply in non-issued cases. Austin and Huxtable have also suggested the use of mediation and clinical ethics committees in conjunction as a possible avenue for conflict resolution, to address concerns around possible oversight of ethical issues in the mediation process.^36^ A further criticism of mediation is that each party may conflate their interests with the best interests of P.^31^ Accordingly, it is important that the mediator has independent access to P, to ensure that P’s voice is heard during the mediation process; although this is invariably more challenging if P is, for example, sedated.

Recognition of mediation as a possible means of dispute resolution resulted in the formation of the Medical Mediation Foundation in 2010.^37^ National Health Service Resolution has also begun to offer mediation in clinical negligence incidents and claims.^38^ Yet further research is needed into the effectiveness of mediation in the medical setting. Brierley *et al*’s review of dispute resolution at Great Ormand Street Hospital (GOSH) reported no instances in which mediation was used, although this is likely due to the fact that mediation was not readily available for healthcare disputes at the time.^39^ Mediation is arguably regarded a last resort,^40^ by which point the dispute has become intractable and both parties have become entrenched in their views. It is worth noting that in this study, discussions around treatment withdrawal between clinicians and parents of critically ill children were successful in nearly 95% of cases^39^; although this may not be representative of dispute resolution overall, as clinicians at GOSH are presumably more accustomed to having difficult conversations about patients with complex medical needs.^36^ In Birchley *et al*’s study of best interests decision-making in paediatric ICU (PICU), PICU clinicians expressed a reluctance to involve the courts, due to the unpredictability of legal outcomes.^41^ This avoidance of external input may result in support being sought too late, therefore worsening the conflict.^40^ Given this inherent reluctance to approach the courts, mediators may have a crucial role as an alternative third-party, earlier in the dispute resolution process.

## Conclusion

Conflict is unfortunately inevitable, hence it is important to set up modelling and tools to minimise conflicts. A deeper understanding of why conflict occurs may allow ICU clinicians to recognise and challenge their own cognitive biases, as well as those of patients’ relatives, thus preventing escalation of conflict when it does occur. When this is not possible, there are options to resolve conflict with external input. While the court is always available as the final arbiter and therefore has its own key place in the conflict resolution sphere, it is not an attractive or practical option for doctors or families. The litigation process is intrinsically adversarial and strains the doctor–patient–relative relationship. Thus, an alternative external party should be considered. Through the establishment of common ground, challenging of cognitive biases and formulation of mutually agreeable solutions; mediation may offer a pragmatic and cost-effective solution to conflict resolution. Mediation is not, however, frequently used and more research is needed into its effectiveness in the intensive care environment.
